# Electronic Medical Record Attitudes and Predictors of Adoption Among Ethiopian Health Professionals: Cross-Sectional Study

**DOI:** 10.2196/63135

**Published:** 2026-03-17

**Authors:** Molawork Ayele Abebe, Besufekad Mulugeta Urgie, Girma Deshimo Lema, Mohammed Awol Yimam, Akine Eshete Abosetugn, Tadesse Mamo Dejene, Asmamaw Abera Kebede, Zenebe Abebe Gebreegziabher, Alemu Kibret Feleke, Deneke Ayele Abebe, Hajira Mohammed Amin, Alemu Basazin Mingude, Zaki A Sherif

**Affiliations:** 1 Department of Internal Medicine, School of Medicine, Debre Berhan University, Debre Berhan, Ethiopia; 2 Department of Epidemiology and Biostatistics, School of Public Health, Debre Berhan University, Debre Berhan, Ethiopia; 3 Department of Admin and Finance, Yekatit-12 Hospital Medical College, Addis Ababa, Ethiopia; 4 Quality and Innovation Directorate, Ministry of Health, Addis Ababa, Ethiopia; 5 Pulse Techs Software Design, Development, and Implementation Company, Addis Ababa, Ethiopia; 6 Department of Nursing, School of Nursing and Midwifery, Debre Berhan University, Debre Berhan, Ethiopia; 7 Department of Biochemistry & Molecular Biology, Howard University College of Medicine, 520 W Street NW, 3430 Adams Building, Washington DC, WA, 20059, United States, 1 202-806-3832

**Keywords:** health professionals, electronic medical record, EMR, EMR implementation, attitude, Ethiopia, communication, patient care, patient health information, developing countries, electronic health record, EHR, predictors

## Abstract

**Background:**

Electronic medical records (EMRs) are increasingly adopted globally to improve health care delivery, yet challenges remain in their acceptance, defined here as favorable attitudes toward their use among health professionals. Understanding factors influencing acceptance is critical for successful implementation.

**Objective:**

This study aimed to identify predictors (or factors) associated with favorable attitudes toward EMRs among health professionals in 3 Ethiopian hospitals.

**Methods:**

A cross-sectional study was conducted from January to March 2025 in 3 Ethiopian hospitals implementing EMRs. A systematic random sampling method was used to initially select 397 health professionals, and data were collected using a structured questionnaire. Multivariate logistic regression was employed to identify predictors of favorable attitudes toward EMRs.

**Results:**

Of the final 382 professionals, 198 (51.8%, 95% CI 0.43-0.53) showed favorable attitudes. Predictors of positive attitude included computer literacy (adjusted odds ratio [AOR] 2.66, 95% CI 1.16-6.09; *P*=.02), EMR training (AOR 2.87, 95% CI 1.80-4.56; *P*<.001), and age of 29 years or younger (AOR 3.05, 95% CI 1.58-5.9; *P*=.001)

**Conclusions:**

Improving computer literacy, providing refresher training, and strengthening management support are key strategies for enhancing health professionals’ attitudes toward EMRs. Future research should explore qualitative insights into barriers and facilitators of EMR adoption.

## Introduction

Electronic medical records (EMRs) represent a significant advancement beyond traditional paper-based records, offering enhanced coordination and communication among health care departments and professionals, ultimately improving the quality of patient care [1]. In contemporary health care settings, EMR systems function as central platforms for interprofessional communication and care facilitation, aligning with governmental mandates for secure collection, storage, and accessibility of patient health information [2,3].

Despite global optimism surrounding EMR systems, their adoption remains limited, especially in resource-constrained regions with high disease burdens [4]. Studies consistently highlight health professionals’ negative attitudes as a primary barrier to EMR adoption in developing countries [5-8]. Challenges such as high acquisition and maintenance costs, lack of financial incentives, limited internet and electrical infrastructure, and insufficient computer skills hinder electronic health record (EHR) adoption [9,10].

Many hospitals in Ethiopia struggle to implement EHR systems for real-time clinical data entry despite efforts to introduce computerized patient health care information systems [11]. The Ethiopian Ministry of Health (MOH) has been working on the development and cascading of health information systems and digital health-related national documents, including the Information Revolution Roadmap II (2020‐2029), the Information Revolution Strategic Plan (2018-2025), and the ICT (Information and Communication Technology) Policy and Digital Health Strategy (2020‐2024) [11]. Although digitalizing the health care system is a key priority of the World Health Organization and the Ethiopian MOH, there are still many challenges to the implementation of EMR [1,4,12]. Lack of funding, capacity, infrastructure issues, legal considerations, resistance to computer technology, computer system literacy, privacy, and confidentiality were the most common barriers to the implementation of EMR in Ethiopia. However, a significant challenge lies in the resistance to change exhibited by many health care professionals. This resistance, often rooted in inadequate knowledge and negative attitudes toward EMR systems, hinders widespread adoption and requires further investigation [11].

Therefore, this study aimed to investigate the attitudes of health professionals in Ethiopia toward EMR implementation in 2023, along with factors influencing those attitudes. The findings may provide valuable insights for health care professionals, health institutions, policymakers, EMR software developers, researchers, and the broader community, and may inform strategies to enhance EMR adoption in Ethiopian hospitals.

## Methods

### Study Design, Setting, and Duration

A multicenter cross-sectional study was conducted to evaluate attitudes and associated factors related to EMR implementation among health professionals in EMR-equipped hospitals in Ethiopia. The study was conducted in 3 selected government hospitals actively using fully integrated EMR systems: Gizawu Hospital, located in Debre Berhan town, North Shoa, Amhara Region; Yekatit 12 Hospital Medical College; and Tirunesh Beijing General Hospital. Data were collected from April 1 to April 30, 2023.

### Study Population, Sample Size, and Sampling Methodology

The study population included health professionals from the 3 hospitals. The total sample size was proportionally allocated to each hospital based on the number of health professionals. A systematic random sampling approach was used to select participants who met the inclusion criterion of work experience of more than 6 months. Individuals on maternity leave, annual leave, sick leave, and those providing voluntary services were excluded. The sample size was determined using the single population proportion formula, accounting for a 5% nonresponse rate [13]. The initial sample size was 397 health professionals.

Study participants were selected using systematic random sampling within each profession. A detailed explanation of the sampling interval calculation, randomization process, and statistical justification is provided in Multimedia Appendix 1.

### Data Collection Instrument and Quality Assurance

A pretested, structured, and self-administered questionnaire was used. The questionnaire was developed based on standard templates derived from a review of published articles [14-18]. Seven data collectors (5 GPs, 2 MPH graduates) and 2 trained GP supervisors monitored the data collection process. A comprehensive pilot study was conducted to ensure instrument validity and reliability. This included content validation by health informatics specialists (CVI=0.81), test-retest reliability (ICC=0.82), and cognitive interviews to refine question phrasing. Full details of the instrument validation, pilot testing, and comparative validation data are available in Table S1 in Multimedia Appendix 1.

### Ethical Considerations 

Ethical approval was obtained from the Ethical Clearance Review Board of the Asrat Woldeyes Health Science Campus, Debre Berhan University (protocol number IRB 01/125/2015), in accordance with the Helsinki Declaration guidelines and regulations. Permission was obtained from the participating hospitals. Written informed consent was obtained from all participants. The authors have maintained the privacy and confidentiality of research subjects’ data and/or identity. The participants of this study were not compensated.

### Data Processing and Analysis

The collected data were entered into EpiData (version 4.6) and exported to SPSS (version 25.0) for analysis. A chi-square test was initially performed, followed by bivariable analysis. Variables with *P*<.25 in the bivariable analysis were included in the multivariable analysis. Adjusted Odds Ratios (AORs) with 95% CI were used to assess associations. Model fitness was evaluated using the Hosmer-Lemeshow test (*P*=.80).

### Dependent and Independent Variables

The dependent variable was attitude toward EMR.

The independent variables were age, computer literacy, knowledge of EMR systems, refresher training on EMR, EMR functionality interruptions, adequate computer access, management support for EMR use, and presence of an ICT center.

### Operational Definition of Terms

The following terms and definitions were used in this study:

- Level of knowledge: Categorized as “Good knowledge” (at or above the mean score) or “Poor knowledge” (below the mean score) based on 12 yes/no questions.
- Attitude: Categorized as “Favorable attitude” (at or above the median score) or “Unfavorable attitude” (below the median score) based on 18 questions.
- Computer literacy*:* Defined as capable of using basic Microsoft Office applications and Internet services.
- EMR training: Refers to refresher training provided to health professionals for at least 1 day twice per year.
- Management support: Indicated by the presence of an EMR committee that conducts regular monitoring and evaluation and takes immediate action whenever problems arise.
- Electric power interruption**:** Defined as experiencing at least one electric power interruption per day.

## Results

### Sociodemographic Characteristics of Study Participants

The study population consisted of health professionals from 3 hospitals, with the total sample size proportionally allocated to each hospital based on the number of health professionals (Table 1). A total of 382 health professionals participated in the study, with a response rate of 96.2%. Nonrespondent profiling was assessed by comparing the demographics of 39 nonrespondents with the sample frame using HER data. No significant differences were found in gender (*P*=.32), profession (*P*=.47), or EMR frequency (*P*=.19). To address concerns about causal inference in this cross-sectional study of EMR implementation attitudes, we implemented a multilayered methodological approach that combines design-based safeguards, advanced statistical techniques, and theoretical triangulation. These included EMR implementation timelines aligned with exposure measurement and hospital-specific rollout periods (6‐18 mo before the survey) using health ministry deployment records. We also captured technology adoption histories through work experience items (eg, “years using digital health tools”) to verify exposure duration.

**Table 1.** Participating hospitals, study population, and sample size.

Name of hospital	Staff, n	Participants, n (%)
Yekatit 12 Hospital Medical College	1265	221 (56)
Tirunesh Beijing Hospital	611	108 (27)
Debre Berhan University, Hakim Gizaw Hospital	380	68 (17)
Total	2256	397 (100)

The majority of respondents were male (n=236, 61.8%). The mean age was 30.04 (SD 4.43) years. Nearly two-thirds of the participants (n=246, 64.4%) had less than 6 years of work experience, and 69.6% (n=266) held a Bachelor of Science degree. A high proportion of study participants (n=339, 88.7%) demonstrated computer literacy, as measured by self-reported proficiency and task completion rates, and 26.6% (n=113) reported previous experience working in private hospitals (Table 2).

**Table 2.** Sociodemographic characteristics of the study participants in electronic medical record (EMR)–implementing hospitals, Ethiopia, 2023 (N=382).

Category	Participants, n (%)
Sex	
Male	236 (61.8)
Female	146 (38.2)
Marital status	
Single	207 (54.2)
Married	175 (45.8)
Type of professional	
Nurse/midwife	144 (37.7)
Pharmacy	36 (9.4)
Laboratory	30 (7.9)
General practitioner	67 (17.5)
Advanced practitioners	44 (11.5)
Radiology	15 (3.9)
Anesthesia	32 (8.4)
Others	14 (3.7)
Work experience	
<6 years	246 (64.4)
≥6 years	136 (35.6)
Educational status	
Diploma	9 (2.4)
BSc	266 (69.6)
MPH/MSc	63 (16.5)
Advanced practitioners	44 (11.5)
Computer literacy	
Yes	339 (88.7)
No	43 (11.3)
Part-time employment in private sector	
Yes	113 (29.6)
No	269 (70.4)

### Knowledge Toward EMR

**Figure 1.**
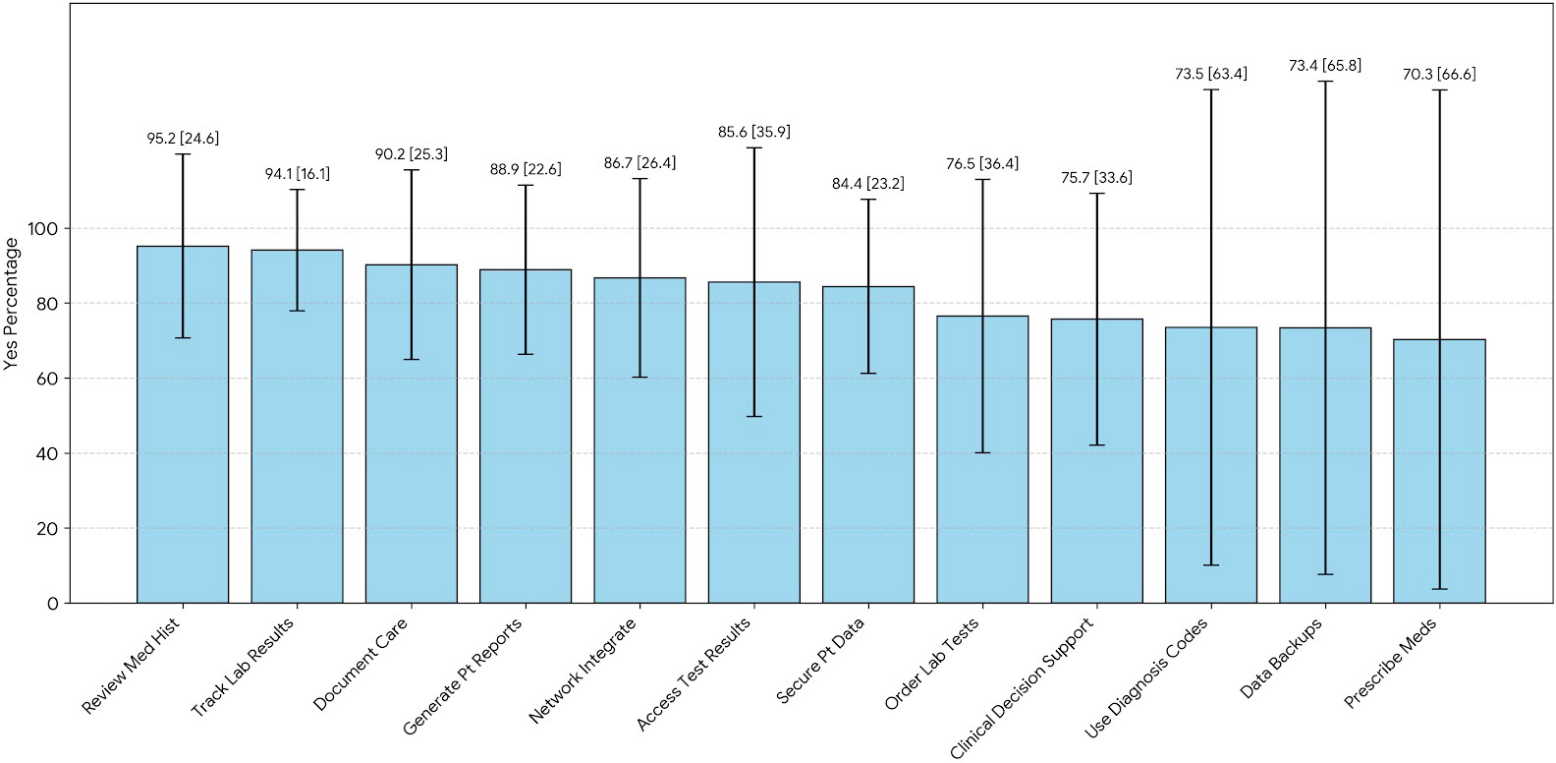
Knowledge of respondents about electronic medical records. The overall proportion of health professionals demonstrating good versus poor knowledge of electronic medical record (EMR) systems among participants in EMR-implementing hospitals in Ethiopia (2023; Table 3). The overall self-reported knowledge of EMR systems, showing that 66% of respondents reported “good knowledge” and 34% reported “Poor knowledge.” Segmented bar chart showing percentage of respondents (in decreasing order of “yes” responses) who reported knowledge of specific EMR parameters, such as “review med hist” and “track lab res.”

Among study participants, 251/382 (65.7%) demonstrated good knowledge of EMR (Table 3, Multimedia Appendix 2). A segmented bar chart showing health professionals’ knowledge of EMR system capabilities with 95% CIs (N=382) is shown in Figure 1. Notably, most respondents were aware of the diverse functions of EMR, including handling patient data, reviewing patient problems, obtaining test results, enabling private ward access, facilitating local network operations, and updating patient information. Furthermore, approximately three-quarters of participants were knowledgeable about EMR functionalities, including laboratory order requests, prescription capabilities, and disease codes, as well as data backup systems (Table 3).

**Table 3.** Health professionals’ knowledge of electronic medical records (EMRs) in EMR-implementing hospitals in Ethiopia, 2023 (N=382).

EMR system capability	Yes, n (%)	No, n (%)
Review patient medical history	364 (95.3)	18 (4.7)
Track patient laboratory results	359 (94)	23 (6)
Document patient care	343 (89.8)	39 (10.2)
Order laboratory tests	292 (76.4)	90 (23.6)
Generate patient reports	339 (88.7)	43 (11.3)
Prescribe medications	268 (70.2)	114 (29.8)
Utilize diagnosis codes	280 (73.3)	102 (26.7)
Access patient test results	327 (85.6)	55 (14.4)
Maintain data backups	280 (73.3)	102 (26.7)
Secure patient data	322 (84.3)	60 (15.7)
Integrate with local network	330 (86.4)	52 (13.6)
Support clinical decision-making	289 (75.7)	93 (24.3)

### Attitude Toward EMR

**Figure 2.**
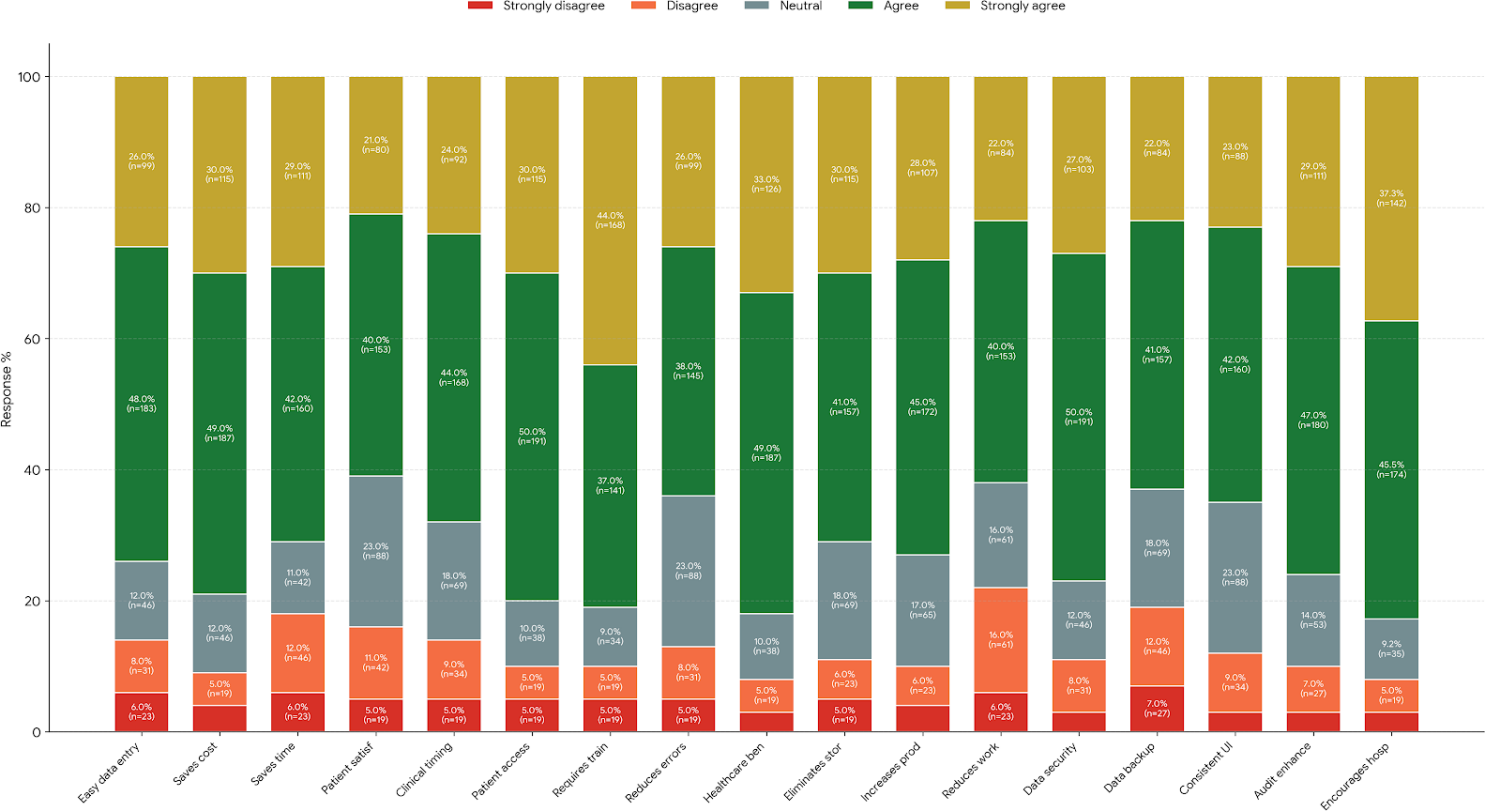
Attitude of respondents toward electronic medical records (EMRs). The distribution of health professionals’ attitudes toward EMR system capabilities. (A) The attitudes of health professionals toward EMR systems showed that 51.8% of professionals had an “Unfavorable” attitude (orange) and 48.2% had a “Favorable” attitude (blue). (B) Stacked bar chart illustrating agreement levels (ranging from “strongly agree” to “strongly disagree”) across 17 perceived benefit dimensions of EMR adoption among health professionals (Table 4). UI: user interface.

**Figure 3.**
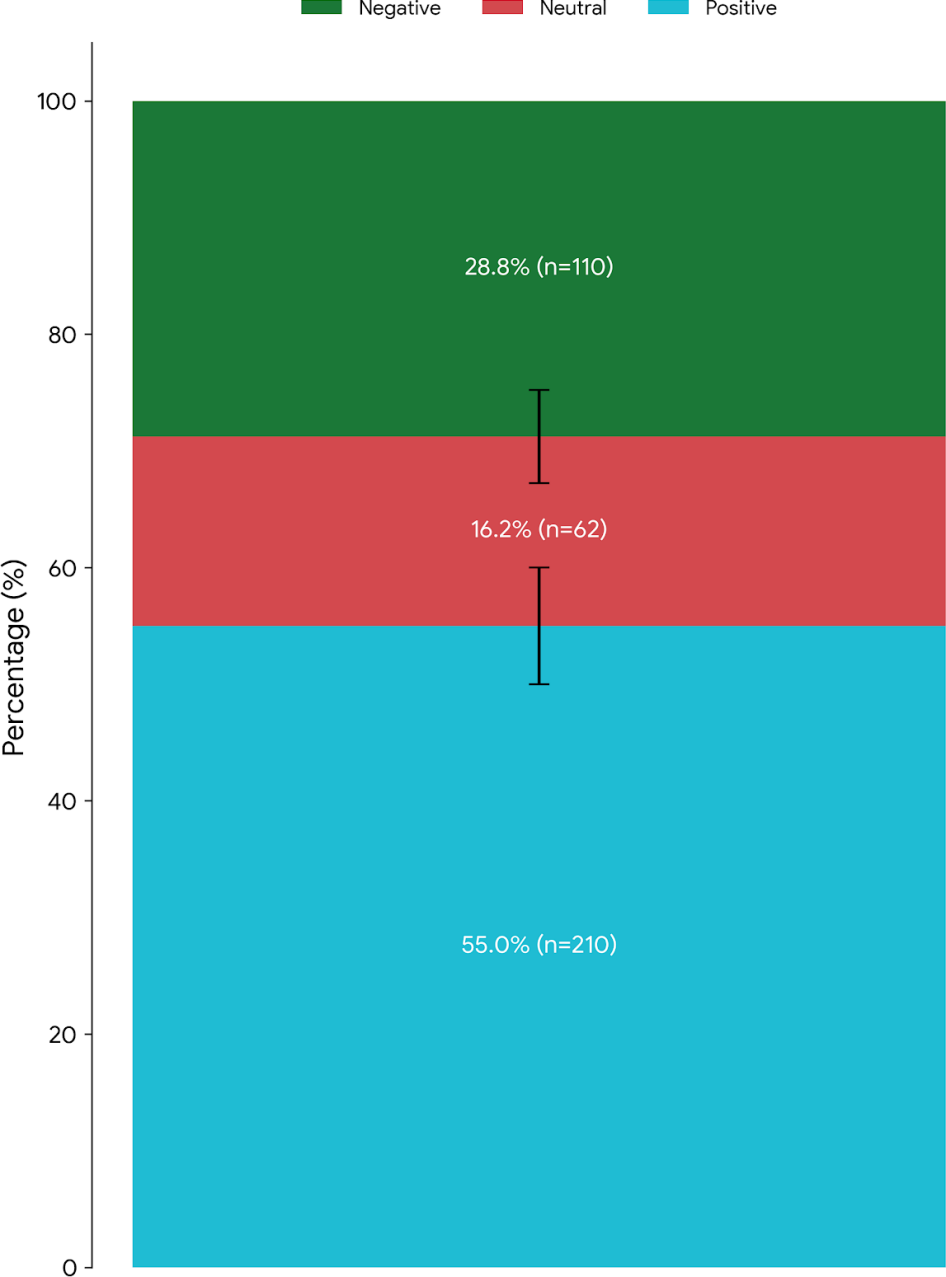
Overall attitudes toward electronic medical records (EMRs) implementation. Distribution of health professionals’ attitudes toward EMR use and implementation across three Likert scale categories: positive (4–5), neutral (3), and negative (1–2). Error bars represent 95% CIs for proportions (n=382). This three-category structure reflects respondents’ sentiments shown in Table 4.

Among the 382 study participants, only 184 (48.2%) demonstrated a favorable attitude towards the implementation of EMR (Multimedia Appendix 3). Please note that Multimedia Appendix 3 intentionally simplifies the data for visualization purposes only. Attitude responses on a 5-point Likert scale were grouped into 3 categories: positive (scores 4‐5), neutral (score 3), and negative (scores 1‐2). Of the 382 respondents, 210 (55%) expressed a positive attitude toward EMRs, 62 (16.2%) had a neutral attitude, and 110 (28.8%) reported a negative attitude. These distributions are detailed in Table 4 and represented in Figure 2. Percentages are accompanied by their absolute values (N=382). Study participants expressed strong agreement on several benefits of EMR, including cost-saving potential (n=117, 30.6%), ease of data entry (n=99, 25.9%), facilitation of timely decisions (n=93, 24.3%), acknowledgment of the need for training (n=166, 43.5%), reduction of medical errors (n=100, 26.2%), enhancement of productivity (n=106, 27.7%), and suitability for audit trials (n=110, 28.8%). Additionally, 156 (40.8%) strongly agreed, and 151 (39.5%) agreed that other hospitals should implement EMR (Table 4). Distribution of health professionals’ overall attitudes toward EMR use across three Likert scale categories: positive (4-5), neutral (3), and negative (1-2) as represented in Figure 3. Error bars represent 95% CIs for proportions (N=382). This 3-category structure reflects respondents’ sentiments and attitudes shown in Table 4.

**Table 4.** Attitudes of health professionals toward use of electronic medical records (EMRs) in EMR-implementing hospitals, Ethiopia, 2023 (N=382).

Perceived EMR benefit	Strongly disagree, n (%)	Disagree, n (%)	Neutral, n (%)	Agree, n (%)	Strongly agree, n (%)
Easy to enter data	23 (6)	28 (7.3)	47 (12.3)	185 (48.4)	99 (25.9)
Saves cost	16 (4.2)	19 (5)	46 (12)	184 (48.2)	117 (30.6)
Saves time	21 (5.5)	46 (12)	42 (11)	161 (42.1)	112 (29.3)
Improves patient satisfaction	20 (5.2)	41 (10.7)	89 (23.3)	152 (39.8)	80 (20.9)
Improves timeliness of clinical decisions	16 (4.2)	35 (9.2)	70 (18.3)	168 (44)	93 (24.3)
Provides easy access to patient information	15 (3.9)	20 (5.2)	39 (10.2)	191 (50)	117 (30.6)
Requires training	21 (5.5)	19 (5)	33 (8.6)	143 (37.4)	166 (43.5)
Reduces the risk of medical errors	18 (4.7)	32 (8.4)	89 (23.3)	143 (37.4)	100 (26.2)
Benefits the health care facility	14 (3.7)	18 (4.7)	38 (9.9)	186 (48.7)	126 (33)
Eliminates need for physical storage	19 (5)	25 (6.5)	69 (18.1)	155 (40.6)	114 (29.8)
Increases productivity	17 (4.5)	23 (6)	66 (17.3)	170 (44.5)	106 (27.7)
Reduces workload	23 (6)	60 (15.7)	62 (16.2)	151 (39.5)	86 (22.5)
Ensures data security	20 (5.2)	22 (5.8)	47 (12.3)	190 (49.7)	103 (27)
Maintains data backup	25 (6.5)	47 (12.3)	72 (18.8)	154 (40.3)	84 (22)
Offers a consistent user interface	17 (4.5)	31 (8.1)	86 (22.5)	160 (41.9)	88 (23)
Enhances audit trail functionality	11 (2.9)	26 (6.8)	55 (14.4)	180 (47.1)	110 (28.8)
Encourages other hospitals to use EMR	14 (3.7)	20 (5.2)	41 (10.7)	151 (39.5)	156 (40.8)

A subgroup analysis regarding patterns of EMR attitudes by age group, profession, and facility type is visualized for all 17 benefit dimensions in Multimedia Appendix 4. We assessed multicollinearity using variance inflation factors (VIF), with all values ranging from 1.1 to 4.7, below the threshold of 5. Moderate correlations (eg, between years of EMR experience and technology self-efficacy) were addressed by combining variables into composites or residualizing overlapping predictors. Robustness checks, including principal component regression and elastic net regularization, confirmed the stability of the results.

### Technology and Organizational Readiness

**Figure 4.**
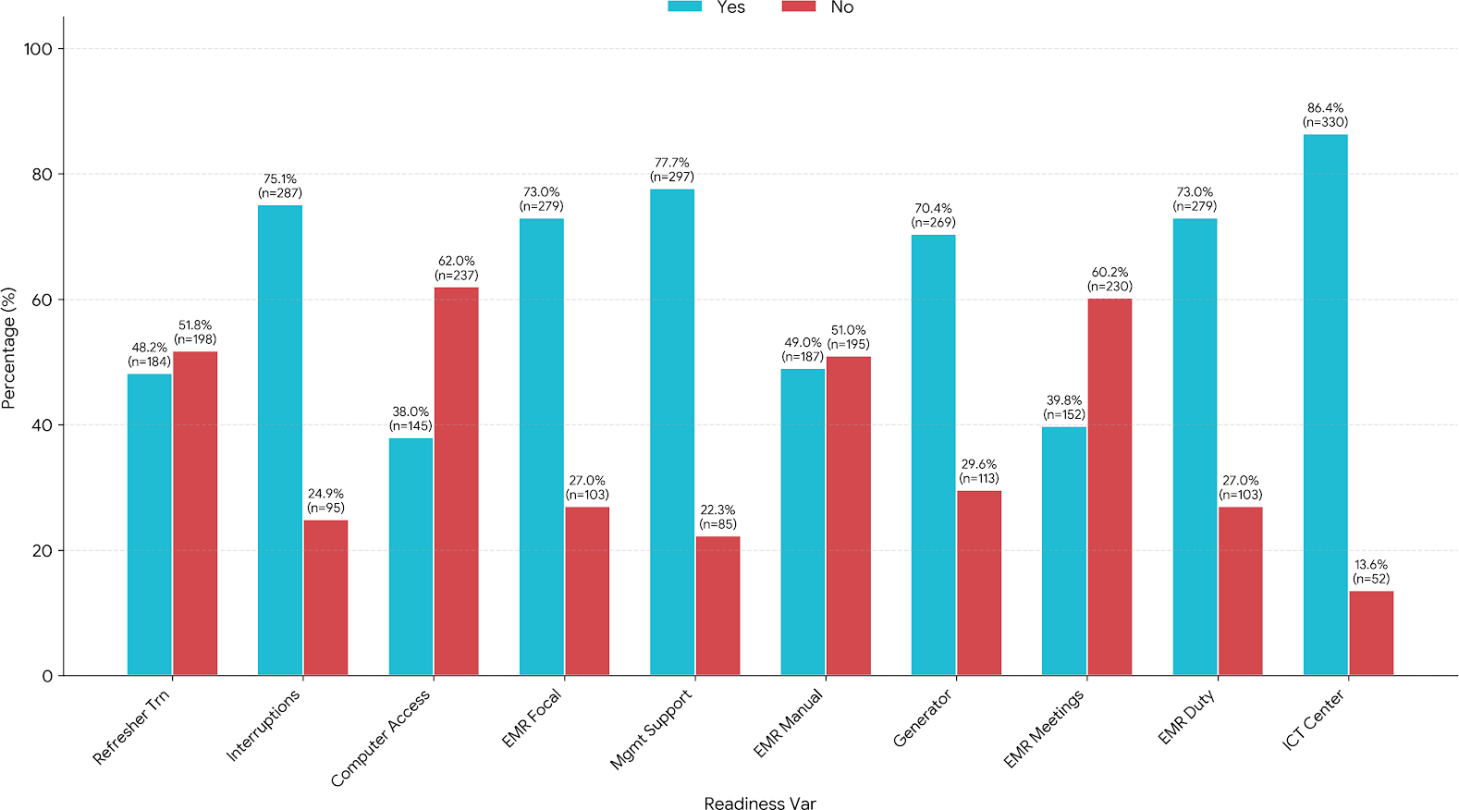
Technological readiness in Ethiopian hospitals. Percentage of “yes” responses (n=382) for technological and organizational readiness in Ethiopian hospitals in 2023. The figure shows the visual comparison of readiness levels across the ten key factors in the dataset. Two bars are displayed for every factor. Yes (%): Percentage of respondents indicating the readiness factor is present or supported; No (%): Percentage of respondents indicating the readiness factor is absent or unsupported (Table 5).

Most respondents reported the fulfillment of certain organizational factors: 73% mentioned the assignment of an EMR focal person and duty person; 77.7% believed that management supports EMR implementation; and 86.4% witnessed the presence of an ICT (Information and Communication Technology) center. However, less than half of respondents received refreshment training on EMR (48.2%); only 38% perceived adequate computer access for EMR, and 39.8% responded that they had regular meetings (Table 5). A clustered bar chart (Figure 4) displays the percentage of health professionals in Ethiopian hospitals who reported technology and organizational readiness for EMR implementation across ten key factors in 2023. For each factor, the proportions of respondents answering “Yes” (indicating presence or support) and “No” (indicating absence or lack of support) are shown side by side. The chart visually highlights areas of strength, such as ICT center availability and management support, as well as gaps, including regular meetings on EMR and refresher training.

**Table 5.** Technology and organizational readiness of health professionals in electronic medical records–implementing Ethiopian hospitals, 2023 (N=382).

Variable	Yes, n (%)	No, n (%)
EMR^a^ refresher training	184 (48.2)	198 (51.8)
Experienced interruption in EMR functionality	287 (75.1)	95 (24.9)
Adequate computer access	145 (38)	237 (62)
Assigned EMR focal person	279 (73)	103 (27)
Management support for EMR	297 (77.7)	85 (22.3)
Presence of EMR manual	187 (49)	195 (51)
Hospital has standby generator	269 (70.4)	113 (29.6)
Regular meeting on EMR	152 (39.8)	230 (60.2)
Hospital has ICT^b^ center	330 (86.4)	52 (13.6)

a EMR: electronic medical record.

b ICT: information and communication technology.

### Advantages of EMR Systems

**Figure 5.**
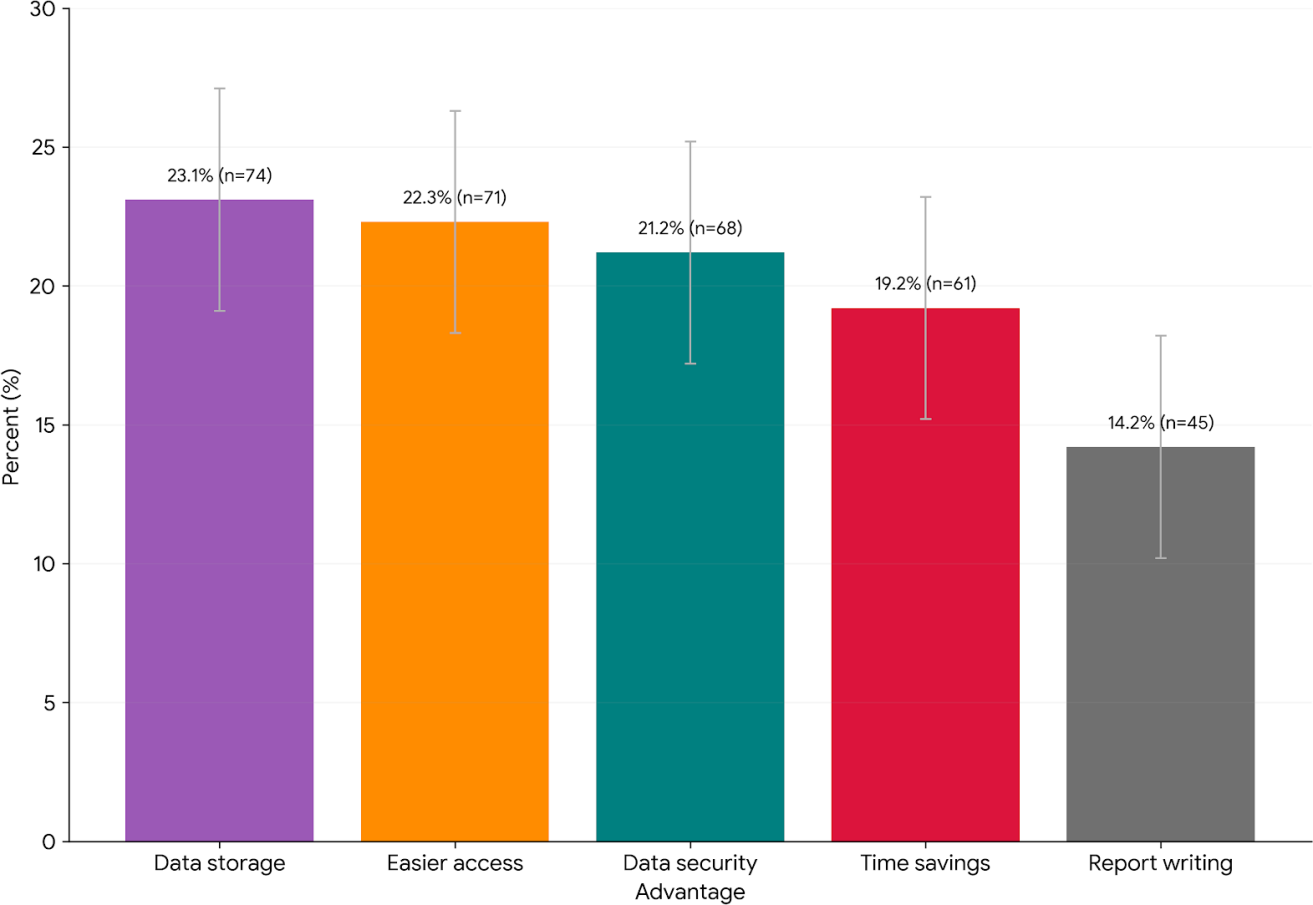
Advantages of electronic medical records (EMRs) systems over paper records. Bar chart of health professionals reported advantages of EMR systems versus paper-based records (n=320). Participants identified time savings, increased storage, easier access to information, simplified report writing, and stronger data security as benefits. Error bars denote 95% CIs for each proportion (Table 4).

The study revealed a strong preference for EMR over paper-based systems among participants. A significant majority (320/382, 83.8%) reported finding EMR systems advantageous over disadvantageous for several reasons (Figure 5). Note that participants could select more than one option.

These advantages included:

- Time savings (19.2%)
- Increased data storage capacity (23.1%)
- Easier access to patient information (22.3%)
- Simplified report writing (14.2%)
- Enhanced data security (21.2%)

### Barriers to EMR Adoption

**Figure 6.**
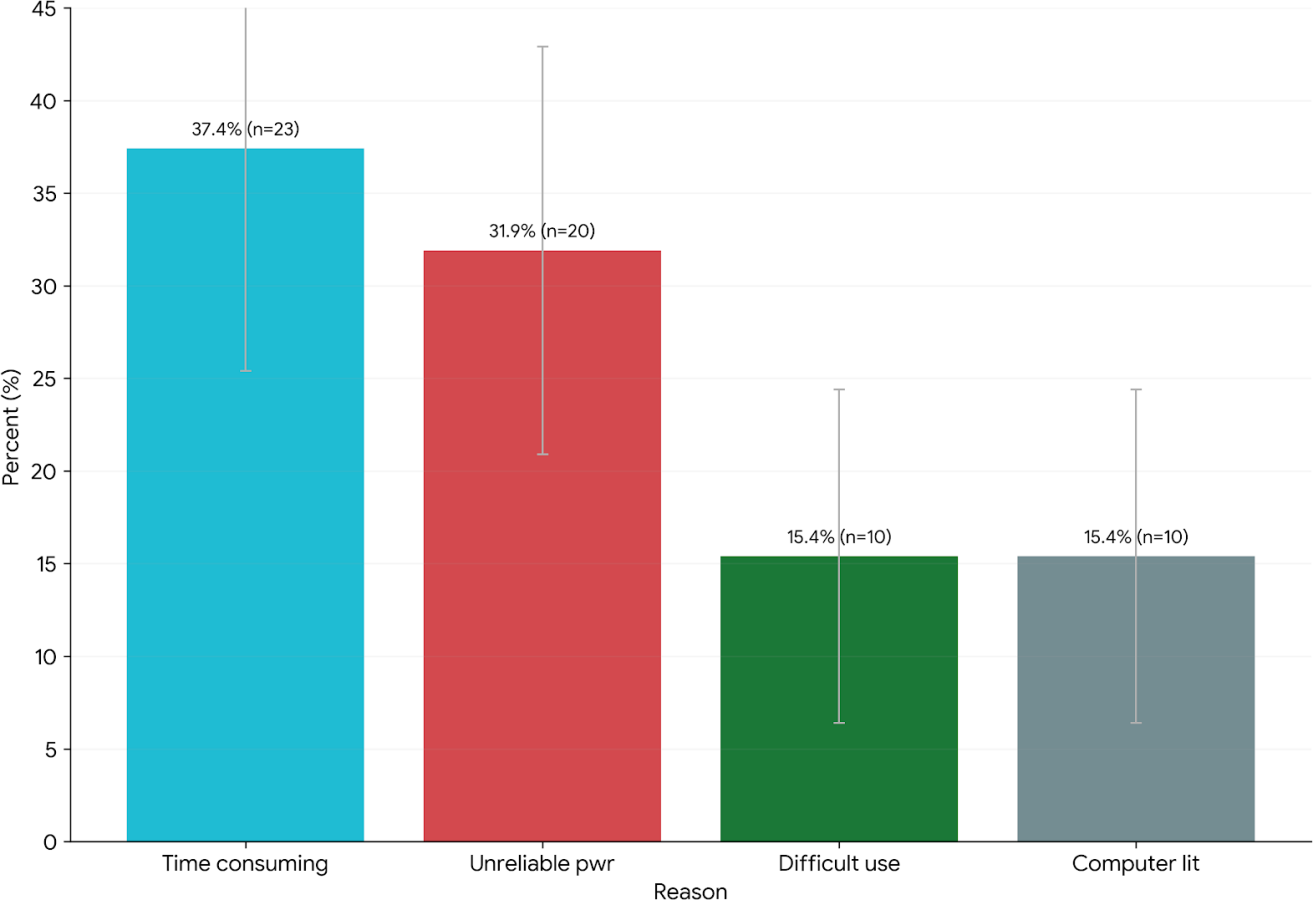
Barriers to adoption of electronic medical records (EMRs). Bar chart of health professionals’ reasons for not preferring EMR systems (n=62) showing percent for each barrier (eg, time, power, usability, computer literacy). Respondents most frequently cited workflow burden, power outage concerns, usability challenges, and limited computer literacy. Error bars denote 95% CIs for each proportion (n=382). Computer lit: computer literacy; Pwr: power grid.

Although most participants favored EMR, some reported challenges. Figure 6 highlights the reasons why a minority of participants expressed reservations about EMR use. The main concerns were the time required for data entry (mentioned as a reason by some who did not prefer EMR). Again, participants can select more than one option.

### Factors Associated With Health Professionals’ Attitudes Toward EMR Systems

All independent variables underwent initial computation using the chi-square test, and variables with a *P* value ≤.05 were selected for bivariate analysis. Among these, variables with a *P* value of ≤.25 were considered for multivariate analysis. Following adjustment for other variables, the multivariable logistic regression analysis revealed that respondents aged ≤29 years were three times more likely to exhibit positive attitudes towards EMR implementation compared to those aged ≥35 years (AOR –3.05; 95% CI −1.58 to 5.9; *P*=.001). Furthermore, individuals who received EMR training were 2.8 times more inclined to have a favorable attitude towards EMR implementation than those without training (AOR −2.87, 95% CI 1.80‐4.56; *P*<.001). Negative signs before AOR values indicate inverse associations (eg, prior poor EMR experience reduces favorability).

The study administered EMR training with role-specific standardization (eg, physicians vs. nurses) but allowed site adaptations for workflow variations. While core curricula were maintained across sites using learning management systems, content delivery mixed future iterations, e-learning, and blended approaches based on staff roles and complexity levels. Health professionals with computer literacy displayed a 2.7 times higher likelihood of holding positive attitudes towards EMR implementation compared to those without computer skills (AOR 2.66; 95% CI 1.16‐6.09), while those with good knowledge of EMR were 1.8 times more likely to have a favorable attitude towards EMR than those with poor knowledge (AOR 1.8; 95% CI 1.10‐2.96) (Table 6).

**Table 6.** Factors associated with health professionals’ attitudes toward electronic medical record (EMR) at three EMR-implementing Ethiopian hospitals, 2023 (N=382).

Variable	Attitude toward EMR, n		COR^a^ (95% CI)	AOR^b^ (95% CI)	*P* value
	Positive	Negative			
Age (years)					
≤29	73	107	2.37 (1.34-4.20)	3.05 (1.58-5.9)	.001
30-34	69	65	1.52 (0.84-2.76)	1.79 (0.92-3.48)	.09
≥35	42	26	1	1	>.99
Computer literacy					
Yes	174	165	3.48 (1.66-7.29)	2.66 (1.16-6.09)	.02
No	10	33	1	1	>.99
Working in private sector					
Yes	66	47	1.80 (1.15-2.80)	1 (0.60-1.69)	.99
No	118	151	1	1	>.99
Knowledge of EMR systems					
Poor	47	84	1	1	>.99
Good	137	114	2.15 (1.39-3.32)	1.80 (1.10-2.96)	.02
Refreshment training on EMR					
Yes	116	68	3.26 (2.15-4.96)	2.87 (1.80-4.56)	<.001
No	68	130	1	1	>.99
EMR functionality interruptions					
Yes	148	139	1.75 (1.09-2.80)	1.43 (0.84-2.46)	.19
No	36	59	1	1	>.99
Adequate computer access					
Yes	82	63	1.72 (1.14-2.61)	0.91 (0.55-1.49)	.70
No	102	135	1	1	>.99
Management support for EMR use					
Yes	157	140	2.41 (1.45-4.01)	1.47 (0.81-2.64)	.20
No	27	58	1	1	>.99
Presence of ICT^c^ center					
Yes	166	164	1.91 (1.04-3.52)	1.26 (0.64-2.49)	.50
No	18	34	1	1	>.99

a COR: crude odds ratio.

b AOR: adjusted odds ratio.

c ICT: information communication technology.

**Figure 7.**
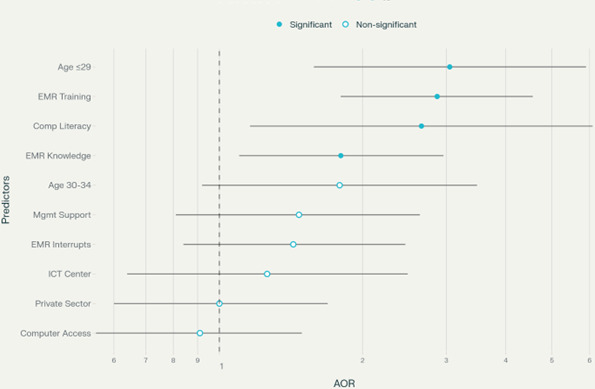
Predictors of positive attitudes toward electronic medical records (EMRs). Forest plot illustrating adjusted odds ratios (AORs) and 95% confidence intervals for predictors of positive attitudes toward EMR use among health professionals in EMR-implementing Ethiopian hospitals (N=382). Each horizontal line represents the 95% CI for an AOR. Variables with AOR values greater than 1 indicate a higher likelihood of a positive attitude toward EMR use. Significant predictors included younger age (≤29 y), computer literacy, refresher training, and good EMR knowledge (*P*<.05). ICT: information and communication technology; Mgmt: management.

The forest plot from the regression analysis (Table 6), showing AORs and CIs for attitude predictors, is represented in Figure 7. The forest plot presents the adjusted odds ratios (AORs) and 95% confidence intervals for predictors of positive attitudes toward electronic medical record (EMR) use among health professionals in EMR-implementing Ethiopian hospitals (N=382). Significant predictors identified in the analysis include younger age (≤29 years), computer literacy, participation in refresher training, and good EMR knowledge (*P*<.05) (Figure 7).

## Discussion

### Principal Findings

EMRs represent a significant advancement beyond traditional paper-based records, offering enhanced coordination and communication among health care departments and professionals, ultimately improving the quality of patient care [1,19]. This study found that just over half (51.8%) of health professionals in the surveyed Ethiopian hospitals reported a favorable attitude toward electronic medical records (EMRs). This rate, while indicating partial acceptance, is significantly lower than that observed in high-income nations like the United States (97%) and other developing countries such as Saudi Arabia (70%) and South Africa (67.2%) [10,17,20]. This disparity likely reflects differences in resource availability, baseline technology access, and national digital health infrastructure.

Our finding is also lower than some previous studies within Ethiopia, with reported favorability rates between 50.6% and 72.8% [16,19,21,22]. This variation may stem from our study’s focus on facilities with full EMR implementation, where staff experience the complete benefits and burdens, unlike settings with partial implementation or where staff are only anticipating a future system.

### Interpretation of Key Predictors

The primary predictors of a positive attitude were computer literacy (AOR 2.66), EMR training (AOR 2.87), and younger age (AOR 3.05). This finding is crucial, as it underscores that EMR acceptance is not passive; it must be actively cultivated through foundational training and skills development.

The strong influence of training and literacy suggests that in the Ethiopian context, fundamental barriers such as the skills to use the system remain the primary challenge. This contrasts with research in contexts like Canada, where factors like collaborative care and financial incentives are more prominent once baseline literacy is assumed [23]. The finding that younger clinicians (“digital natives”) are more receptive aligns with prior adoption models [16,22,24]. This highlights an urgent need for established, experienced professionals who may be less familiar with digital systems but whose buy-in is critical for successful implementation.

Interestingly, factors like management support, computer access, and the presence of ICT centers were not significantly associated with attitudes in our model. This finding contrasts with other studies in the Amhara region and Dire Dawa [5,21,22]. This discrepancy may suggest that while access to hardware is a prerequisite, it is not sufficient to foster positive attitudes. Rather, the skills and training to use that hardware effectively are what drive professional acceptance [16,23].

### Policy and Management Implications for Ethiopia’s Digital Strategy

Our findings generate specific, actionable recommendations for Ethiopia’s digital health strategy.

1. Mandate Standardized Training: The strong link between training and attitude (AOR 2.87) suggests that ad-hoc training is insufficient. We recommend a national, role-specific blended training program that combines e-learning with hands-on simulations and is reinforced by annual competency checks.
2. Invest in Foundational Skills: Computer literacy was an even stronger predictor than EMR training. This implies that EMR training will fail if staff lack basic computer skills. We recommend that hospitals form partnerships with local institutions to provide foundational computer skills workshops for all staff.
3. Develop Peer-Coach Programs: To support older or more resistant professionals, we recommend establishing peer-coach certification programs. Training “clinician informaticists” to provide on-site, department-specific mentoring can effectively address resistance and improve adoption.
4. Integrate EMR Competency: To ensure long-term proficiency and signal a systemic commitment, EMR competency requirements could be integrated into national licensure renewal processes.

### Limitations and Future Research

This study has several limitations. The sample was drawn from urban hospitals, which may limit generalizability to rural settings with different infrastructure. Furthermore, the cross-sectional design is a snapshot in time and cannot establish cause-and-effect relationships; professionals with a positive attitude may be more likely to seek out EMR training, rather than the other way around.

To address these limitations, future research should employ qualitative methodologies. Longitudinal ethnography could track EMR adoption over time to identify evolving attitudes. We also recommend Participatory Action Research (PAR) to co-design training protocols with frontline staff, which can foster stakeholder buy-in. Finally, narrative inquiry—collecting stories from both "resistors" and "early adopters"—could reveal deeper, more nuanced experiential perceptions of EMR utility that our survey may have missed.

### Conclusions

In this study, nearly half of the respondents had low attitudes towards the implementation of EMR. Variables related to socio-demographic (health professionals’ age), personal (health professionals’ knowledge of EMR), technology-related (health professionals’ computer literacy), and organization-related (health professionals’ EMR training) factors were significantly associated with health professionals’ attitudes towards the EMR system.** **

### Recommendations

Based on the findings of this study, it is important to focus on the gaps identified to implement EMR systems efficiently and effectively in all Ethiopian hospitals. Enhancing health professionals’ attitudes and contextualizing EMR training in the health care system are highly recommended for scaling up EMR use. For future researchers interested in EMR-related research in Ethiopia**,** employing mixed methods (quantitative and qualitative) could be highly beneficial.

## Supplementary material

10.2196/63135 Multimedia Appendix 1 Methodological details.

10.2196/63135 Multimedia Appendix 2 Knowledge of respondents.

10.2196/63135 Multimedia Appendix 3 Attitudes toward electronic medical record use.

10.2196/63135 Multimedia Appendix 4 Distribution of electronic medical record attitudes by demographic and professional subgroups.

## Abbreviations

AOR: adjusted odds ratio
COR: crude odds ratio
EHR: electronic health record
EMR: electronic medical record
HIS: health information system
HMIS: health management information system
ICC: intraclass correlation coefficient
ICT: information and communication technology
MOH: Ministry of Health
SRS: systematic random sampling
