# Supplementary material for: Electronic Medical Record Attitudes and Predictors of Adoption Among Ethiopian Health Professionals: Cross-Sectional Study
Source: JMIR Form Res. 2026 Mar 17;10:e63135. doi: 10.2196/63135 (PMC12994757; doi:10.2196/63135)
Supplement: Multimedia Appendix 1 [file formative-v10-e63135-s001.docx]

**Multimedia Appendix 1**. **Full details of the instrument validation, pilot testing, and comparative validation data are shown**.

**Methodological Details**

**1. Sampling Interval and Selection Process**

The sampling interval (k) was calculated for each hospital and professional group using the formula: k = N/n, where: N is the total number of eligible health professionals (i.e., 1265, 611, and 380 in Yekatit, Tirunesh, and Hakim, respectively) in each hospital and n is the required sample size from that hospital. For example, if Hakim Gizawu Hospital had 380 eligible health professionals as presented in Table 1, and needed to contribute 68 participants, the sampling interval would be k = 380/68 ≈ 5.6.

To select participants, we used a computerized random number generator to choose a number between the starting point (1) and the calculated sampling interval (k) for each professional category within each hospital, ensuring a random starting point. To address potential concerns about periodicity in the sampling frame, we carefully examined the organization of staff lists before applying systematic sampling to ensure no cyclical patterns existed. Staff lists were arranged alphabetically rather than by any characteristic that might correlate with attitudes toward EMR.

We strengthened the statistical justification for our final sample size of 382 by providing sufficient statistical power (80%) to detect meaningful differences. The proportional allocation strategy ensures that each hospital's contribution to the sample is representative of its size, maintaining the sample's self-weighting and minimizing the need for complex weighting procedures during analysis.

To improve documentation of the actual sampling process, we documented each step: for each hospital and professional category, we recorded the total eligible population, the required sample size, the calculated sampling interval, the randomly selected starting point, and the resulting selection sequence.

**2. Instrument Validity and Pilot Testing**

To address instrument validity, we enhanced documentation of reference-specific validation studies from cited instruments and reported Content Validity Index (CVI) scores from expert reviews. The instrument underwent content validation through expert review (n=2 health informatics specialists) using a 4-point Likert scale for relevance. Items achieving CVI ≥0.78 were retained.

A pilot study was conducted with 40 health professionals (10% of the final sample) from non-participating wards, ensuring representation across professions and experience levels. Test-retest reliability was assessed with 15 participants at 2-week intervals (ICC=0.82 for the attitude scale). Cognitive interviews revealed difficulties with the 'EMR functionality interruptions' item, which was rephrased to 'daily EMR downtime duration' based on pilot feedback. Response consistency improved from 68% to 92% after simplifying Likert-scale anchors following a pilot analysis.

We included a Comparative Validation Table (Table S1) to strengthen our methodology.

**Table S1. Comparative Validation Data**

| **Validation Metric** | **Current Study** | **Yilma et al. [5]** | **Kalayou et al. [14]** |
| --- | --- | --- | --- |
| Content Validity Index | 0.81 | 0.79 | 0.83 |
| Attitude Scale @ | 0.78 | 0.76 | 0.81 |
| Test-Retest ICC | 0.82 | 0.75 | 0.84 |

==
