# Supplementary figures and images for: Electronic Medical Record Attitudes and Predictors of Adoption Among Ethiopian Health Professionals: Cross-Sectional Study
Source: JMIR Form Res. 2026 Mar 17;10:e63135. doi: 10.2196/63135 (PMC12994757; doi:10.2196/63135)

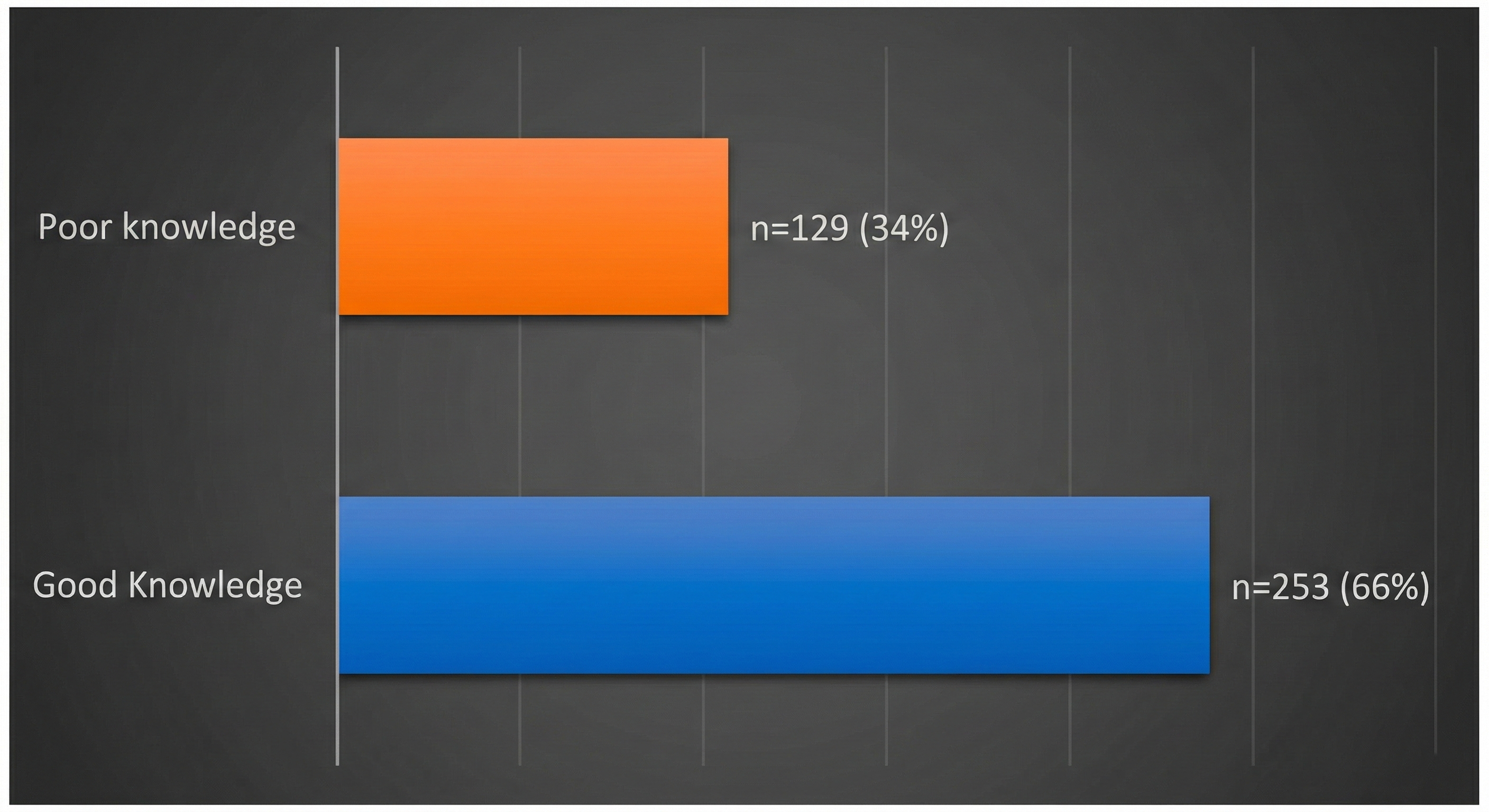

Supplement: Multimedia Appendix 2 [file formative-v10-e63135-s002.png]

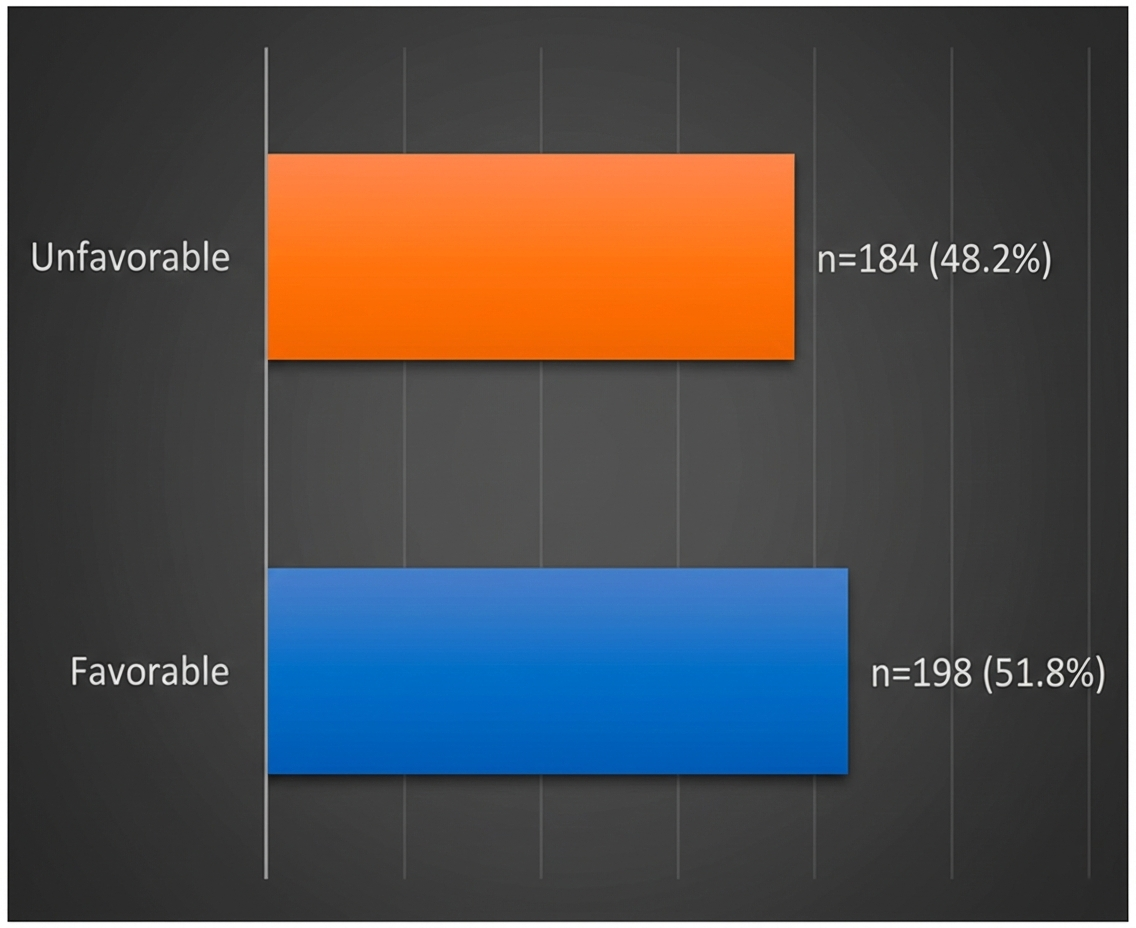

Supplement: Multimedia Appendix 3 [file formative-v10-e63135-s003.png]

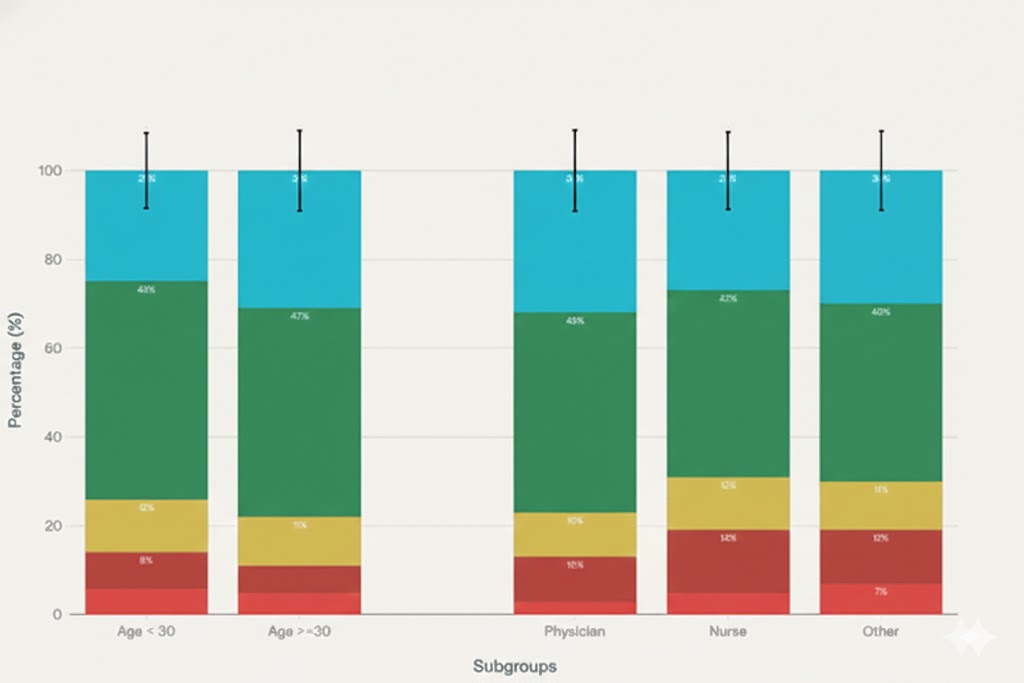

Supplement: Multimedia Appendix 4 [file formative-v10-e63135-s004.jpg]
